# ANK2 as a novel predictive biomarker for immune checkpoint inhibitors and its correlation with antitumor immunity in lung adenocarcinoma

**DOI:** 10.1186/s12890-022-02279-2

**Published:** 2022-12-20

**Authors:** Wengang Zhang, Xiaoling Shang, Ni Liu, Xinchun Ma, Rui Yang, Handai Xia, Yuqing Zhang, Qi Zheng, Xiuwen Wang, Yanguo Liu

**Affiliations:** Department of Medical Oncology, Qilu Hospital of Shandong University, 107 Wenhuaxi Road, Jinan, 250012 Shandong China

**Keywords:** ANK2, Lung adenocarcinoma, Immune checkpoint inhibitors, Biomarker

## Abstract

**Background:**

Immune checkpoint inhibitors (ICIs) have been shown to significantly improve the survival of patients with advanced lung adenocarcinoma (LUAD). However, only limited proportion of patients could benefit from ICIs. Novel biomarkers with strong predictability are needed for clinicians to maximize the efficacy of ICIs. Our study aimed to identify potential biomarkers predicting ICIs efficacy in LUAD.

**Methods:**

The Cancer Genome Atlas (TCGA) PanCancer Atlas studies in cBioportal were used to evaluate the mutation frequency of ANK2 across multiple cancers. Clinical and mutational data for LUAD from ICIs-treated cohorts (Hellmann et al. and Rizvi et al.) were collected to explore the correlation between ANK2 mutation and clinical outcomes. In addition, the relationship between ANK2 expression and clinical outcomes was analyzed using LUAD data from TCGA and Gene Expression Omnibus. Furthermore, the impact of ANK2 mutation and expression on the tumor immune microenvironment of LUAD was analyzed using TCGA and TISIDB databases.

**Results:**

Patients with ANK2 mutation benefited more from ICIs. In ICIs-treated cohort, prolonged progression-free survival (PFS) (median PFS: NR (not reached) vs. 5.42 months, HR (hazard ratio) 0.31, 95% CI 0.18–0.54; P = 0.0037), improved complete response rate (17.65% vs. 1.85%, P = 0.0402), and improved objective response rate (64.71% vs. 24.07%, P = 0.0033) were observed in LUAD patients with ANK2 mutation compared to their wild-type counterparts. Regarding ANK2 expression, it was observed that ANK2 expression was decreased in LUAD (P < 0.05) and a higher level of ANK2 expression was associated with longer overall survival (HR 0.69, 95% CI 0.52–0.92; P = 0.012) in TCGA LUAD cohort. Moreover, ANK2 mutation or higher ANK2 expression correlated with enhanced antitumor immunity and “hot” tumor microenvironment in LUAD, which could be potential mechanisms that ANK2 mutation facilitated ICIs therapy and patients with higher ANK2 expression survived longer.

**Conclusion:**

Our findings suggest that ANK2 mutation or increased ANK2 expression may serve as a favorable biomarker for the efficacy of ICIs in patients with LUAD.

**Supplementary Information:**

The online version contains supplementary material available at 10.1186/s12890-022-02279-2.

## Background

Lung cancer is the leading cause of cancer-related deaths [1], among which lung adenocarcinoma (LUAD) being the most prevalent subtype [2]. Given the fact that the majority of LUAD patients are diagnosed at an advanced stage, surgery is improbable and chemotherapy has historically been the primary treatment option [3]. However, the 5-year survival rate is less than 5% for advanced LUAD patients receiving chemotherapy [4]. Recently, the introduction of immune checkpoint inhibitors (ICIs) is a major breakthrough in LUAD treatment, which has improved the 5-year survival rate of advanced LUAD from less than 5% in the era of chemotherapy to approximately 30% currently for patients lacking sensitizing EGFR (epidermal growth factor receptor) or ALK (anaplastic lymphoma kinase) mutations [5]. Nevertheless, in most scenarios, only 20–40% of patients respond to ICI treatment and even fewer achieve long-term disease remission [6, 7], making the identification of biomarkers for patients likely to respond to ICIs therapy a critical step in identifying the candidate population.

Notably, PD-L1 expression on tumor cells or immune cells, as well as tumor mutation burden (TMB), can predict the therapeutic benefit of ICIs, with ICIs being more efficacious in prolonging the survival of patients with higher PD-L1 expression or higher TMB [8, 9]. Consequently, PD-L1 expression and TMB are approved as biomarkers in predicting efficiency of ICIs. However, certain limitations exist in these biomarkers. KEYNOTE-001 showed that the response rate to ICIs was 45.2% for those with PD-L1 expression ≥ 50% [10], whereas prolonged survival was also observed for patients with PD-L1 < 1% [11], suggesting that PD-L1 alone as a predicting biomarker would miss some responders and include some non-responders. In addition, inter-assay and intra-tumoral heterogeneity are identified, severely affecting the sensitivity and specificity of its predictability [12, 13]. TMB encounter similar concerns. Therefore, novel biomarkers with powerful predictability are urgently needed to optimize the treatment strategies of ICIs.

Unlike lung cancer of other histological types, advanced LUAD is characterized with higher prevalence of driver mutations, which determines the therapeutic strategy [14]. Representatively, targeted therapy is considered the first choice for patients with EGFR mutation or ALK mutation due to their superior performance [14]. Notwithstanding, gene mutations also have dramatic impacts on the efficacy of immunotherapy. Clinically, EGFR or ALK mutations are discovered to weaken the efficacy of immunotherapy [15, 16]. Likewise, patients harboring KEAP1 or STK11 mutations benefit less from immunotherapy compared to those with wild-type [17]. In contrast, LRP1B mutation [18], NOTCH4 mutation [19], ZFHX3 mutation [20], EPHA7 mutation [21], SETD2 mutation [22] and POLE/POLD1 mutation [23] were proven to be favorable biomarkers for ICIs treatment. Therefore, genetic mutations in LUAD play vital roles in regulating the sensitivity of ICIs treatment. Elucidating the relationship between key genetic mutations and ICIs efficacy will provide crucial evidence for distinguishing beneficiary patients.

Through analysis of the cBioportal database, we found that Ankyrin-B (ANK2) exhibited a higher mutation frequency in LUAD. The Ankyrins, belonging to the adapter protein family, consist of Ankyrin-R (ANK1), Ankyrin-B (ANK2), and Ankyrin-G (ANK3) [24]. Initially, ANK2 was considered as a key cytoskeletal-associated protein in the brain [25]. Besides, it was discovered as a pivotal adaptor and scaffolding protein in the heart [26]. Remarkably, recent studies have demonstrated that Ankyrins contribute to the modulation of various biological activities in cancers. Ying and his colleagues proved that ANK2 was overexpressed in pancreatic ductal adenocarcinoma and inhibition of ANK2 reduced invasion ability of pancreatic cancer cells [27]. Moreover, vital roles of Ankyrins have been observed in gastric cancer, breast cancer, and prostate cancer [28–30]. However, the impact of ANK2 (ANK2 mutation and expression) on the prognosis of LUAD patients, especially those treated with ICIs, is unknown.

In this study, the relationship between ANK2 mutation and ICIs efficacy in LUAD was analyzed through systematically collected and integrated clinical and genomic data from the cBioportal database (Hellmann et al. and Rizvi et al. cohorts). Besides, the prognostic value of ANK2 expression in LUAD was evaluated using the Cancer Genome Atlas (TCGA) database. Gene Expression Omnibus (GEO) datasets were used for further validation. Moreover, we analyzed the impact of ANK2 mutation or expression on immune-related signatures in tumor immune microenvironment (TIME) using TISIDB.

## Materials and methods

### Data collection

**Fig. 1:**
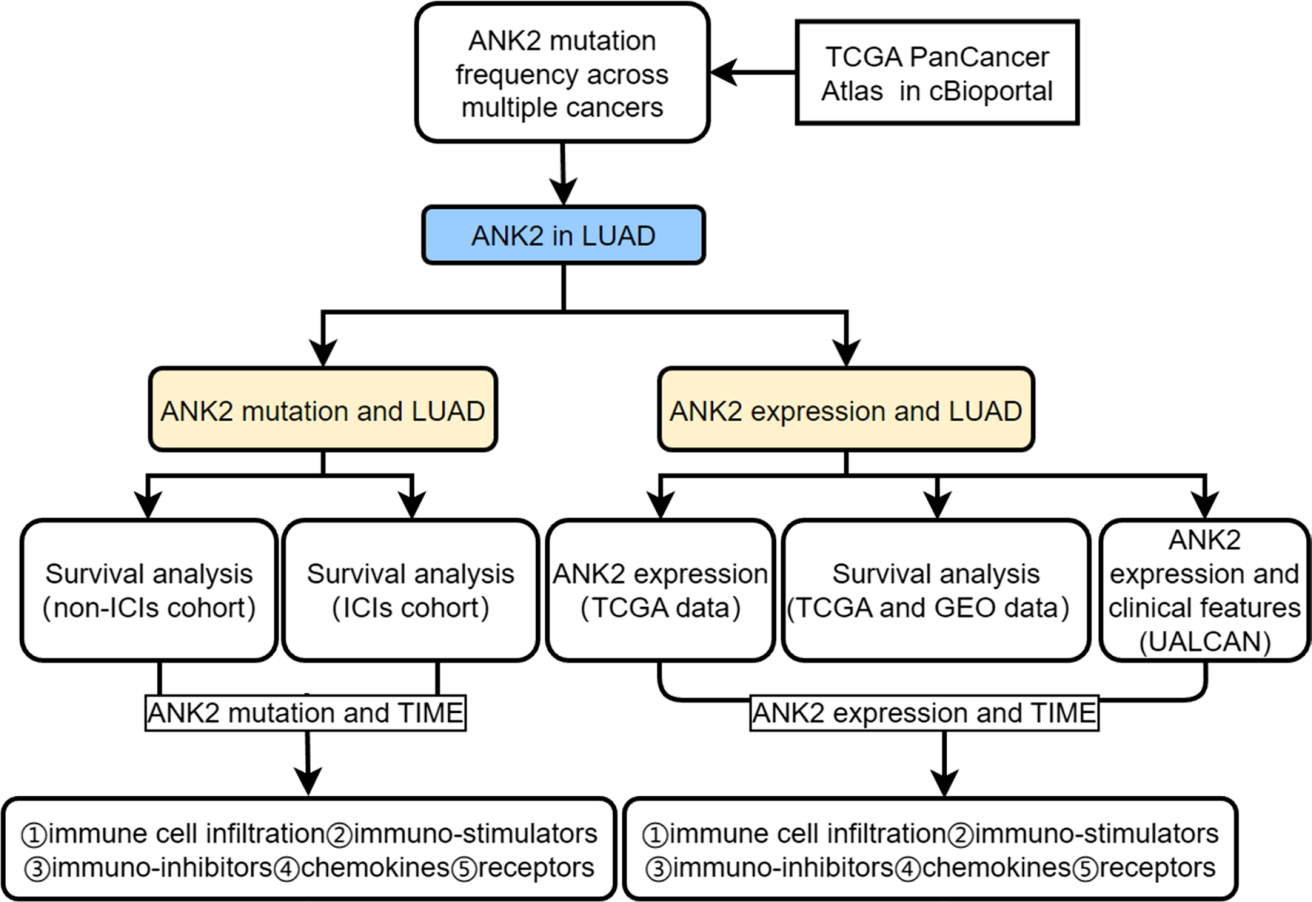
The flowchart of the study

The flowchart of our study was showed in Fig. 1. A total of 10,967 samples from the TCGA PanCancer Atlas studies in cBioportal (https://www.cbioportal.org/) [31] were selected to evaluate the mutation frequency of ANK2 across multiple cancers. 12 LUAD studies in cBioportal totaling 4309 samples were used to perform survival analysis [31]. Moreover, the clinical and mutational data of ICIs-treated lung cancer cohort in cBioportal (Hellmann et al. and Rizvi et al.) [32, 33] were downloaded in order to investigate the effect of ANK2 mutation on ICIs efficacy for LUAD patients. Samples with histology adenocarcinoma were included. After excluding 17 squamous samples, 74 LUAD samples were eventually included as the ICIs-treated cohort. LUAD harboring ANK2 nonsynonymous somatic mutations was defined as ANK2-mutant (**ANK2-MT**), otherwise, it was defined as ANK2 wild type (**ANK2-WT**). Furthermore, RNA-seq and clinical data of LUAD were downloaded from TCGA (www.tcga-data.nci.nih.gov/tcga) to explore the influence of ANK2 expression on the survival of LUAD patients [34].

### ANK2 mutation and outcomes of LUAD receiving ICIs

ICIs-treated cohort was used to analyze the relationship between ANK2 mutation and progression-free survival (PFS) in LUAD patients, as well as the response to ICIs treatment. The response to ICIs was evaluated according to the Response Evaluation Criteria in Solid Tumors (RECIST) 1.1. Durable clinical benefit (DCB) was defined as complete response (CR), partial response (PR) or stable disease (SD) lasting over six months. All other patients were considered to have no durable benefit (NDB). The objective response rate (ORR) was defined as the percentage of patients with CR or PR. Besides, we investigated the association between ANK2 mutation and multiple existing biomarkers for ICIs, including TMB, predicted neoantigen burden (PNB), and PD-L1 expression levels, using ICIs-treated cohort. TMB was defined as the number of nonsynonymous alterations (SNVs or indels) per patient. PD-L1 expression levels on tumor cells or infiltrating immune cells of < 1%, 1–49%, ≥ 50%, were considered negative, weak, and strong, respectively. Moreover, co-mutations of ANK2 with other genes, such as DNA damage repair (DDR)-related genes and mismatch repair (MMR)-related genes, etc., were calculated with 12 LUAD studies from cBioportal.

### ANK2 expression in LUAD

The CPTAC pan cancer samples in UALCAN database (http://ualcan.path.uab.edu/) were used to explore ANK2 expression levels across various cancers [35]. Then, the downloaded LUAD data from TCGA were employed to compare ANK2 expression between LUAD and healthy tissues.

### RNA extraction and quantitative-PCR (q-PCR)

Q-PCR was further performed to validate the difference in ANK2 expression between LUAD and healthy tissues. In detail, total RNA was isolated from healthy lung epithelial cell line (BEAS-2B) and lung adenocarcinoma cell lines (PC9, H1975, A549 and HCC827) using Trizol reagent and cDNA was synthesized according to the manufacturer’s protocol (Accurate Biology, China). Next, q-PCR was performed using SYBR® Green Premix Pro Taq HS qPCR kit (Accurate Biology, China). The mRNA levels were normalized to that of β-actin based on the threshold cycle of each sample. Primer sequences were as follows:

ANK2 forward primer: CGCTAGGAAAGACGACACCA,

ANK2 reverse primer: CCACTCTCAGTTGTCCTATTCAC,

β-actin forward primer: GAAGAGCTACGAGCTGCCTGA,

β-actin reverse primer: CAGACAGCACTGTGTTGGCG.

### Survival analyses based on ANK2 expression in LUAD patients

Based on median ANK2 expression, TCGA LUAD samples were divided into two groups, high ANK2 expression group and low ANK2 expression group. Next, the Kaplan–Meier survival curves were depicted utilizing the survival package [36]. Ten LUAD cohorts (GSE11969, GSE13213, GSE26939, GSE29013, GSE30219, GSE31210, GSE37745, GSE42127, GSE50081, GSE72094) from GEO were utilized to validate the relationship between ANK2 expression and patient survival. Moreover, the relationship between ANK2 expression and clinicopathological parameters (including tumor stage, tumor grade, and various pathway alterations) in LUAD was investigated through UALCAN database (http://ualcan.path.uab.edu/) [35].

### Correlation analysis of immune characteristics

The infiltration levels of 22 tumor-infiltrating lymphocyte subsets between ANK2-MT and ANK2-WT were evaluated using LUAD data from TCGA by CIBERSORT algorithm (https://cibersort.stanford.edu/) [37]. Single-sample GSEA (ssGSEA) with the “GSVA” R package was employed to quantify the infiltration scores of 15 immune cells between the low- and high-ANK2 expression group using TCGA LUAD data [38, 39]. TIMER database was used to further validate the correlation between ANK2 (ANK2 expression or ANK2 mutation) and immune infiltrating cells [40]. The impact of ANK2 copy number variation (CNV) on immune cell infiltration of LUAD was explored by TIMER as well [40]. Further, we evaluated the association between ANK2 expression and gene markers of various immune cells in LUAD using TIMER [40]. The gene markers of immune cells were determined through R&D Systems (www.rndsystems.com/cn/resources/cell-markers/immune-cells). The association of ANK2 with varying immune characteristics (immuno-stimulators, immuno-inhibitors, major histocompatibility complex (MHC) molecules, chemokines, and their receptors) in LUAD was evaluated using TISIDB (http://cis.hku.hk/TISIDB/index.php) [41], an integrated repository portal for tumor-immune system interactions.

### Statistical analyses

The Kaplan–Meier method was applied to depict survival curves, and log-rank test was applied to evaluate P values. ANK2 expression between LUAD and healthy tissues was compared utilizing Mann–Whitney U test. The q-PCR results were analyzed by One-way ANOVA test. The association between ANK2 mutation and clinicopathological parameters (such as PD-L1, CR, PR, SD, PD, ORR, DCB) was analyzed through Pearson chi-squared test or Fisher’s exact test. The relationship between ANK2 mutation and TMB or PNB was analyzed by Mann–Whitney U test. All analyses in our study were conducted through R software (version 3.6.3), GraphPad Prism (version 9.0), Stata (version 16) or online databases (TIMER, UALCAN, TISIDB, cBioportal). Statistical difference was considered when P < 0.05.

## Results

### ANK2 mutation frequency and its correlation with survival of patients with LUAD

**Fig. 2:**
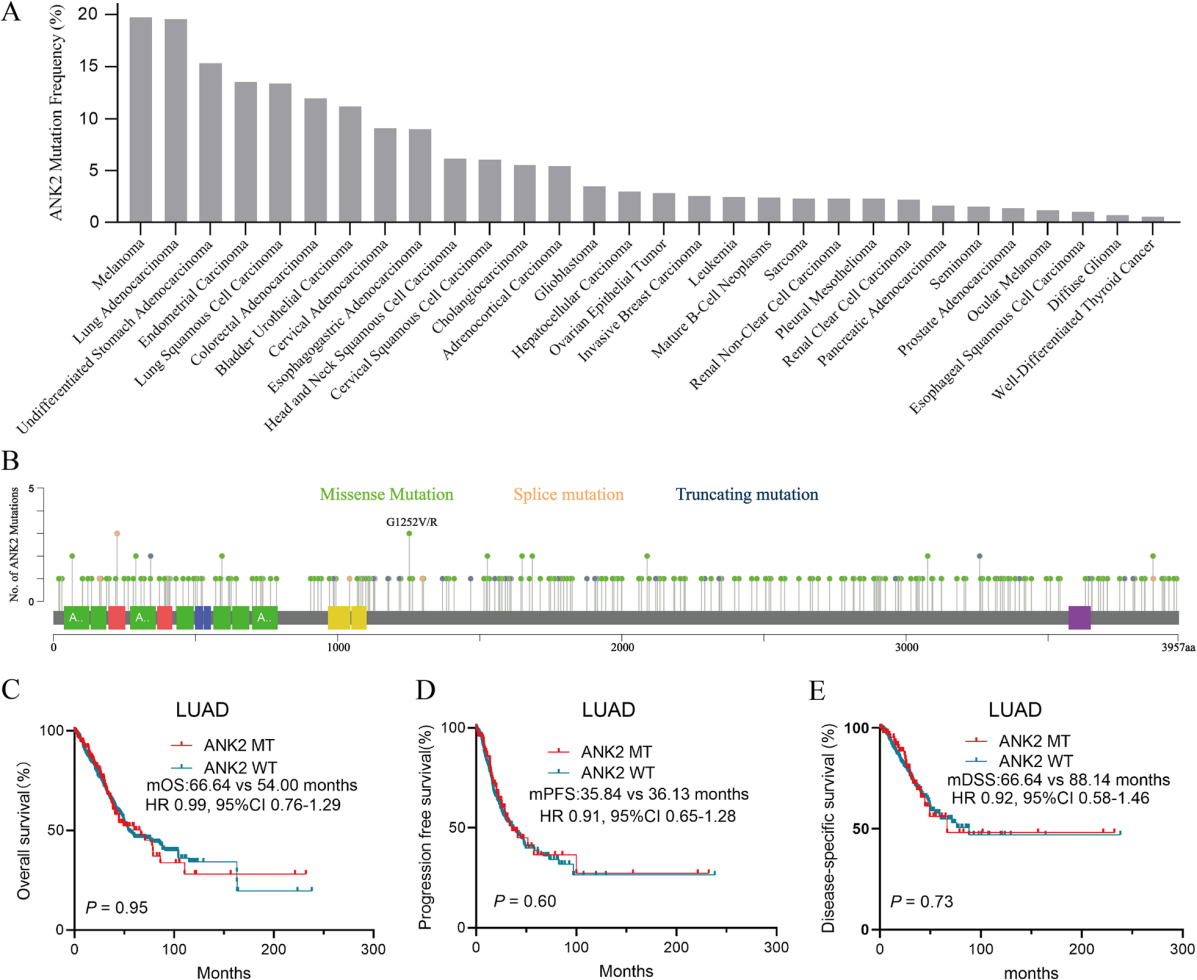
ANK2 mutation frequency across cancers and survival analysis of LUAD based on ANK2 mutation status. **A**: The prevalence of ANK2 mutation across 30 types of cancers. **B**: Lollipop plot shows the distribution of ANK2 mutation in LUAD. **C**: Overall survival (OS) analysis stratified by ANK2 mutation status in LUAD from cBioportal. **D**: Progress-free survival (PFS) analysis stratified by ANK2 mutation status in LUAD from cBioportal. **E**: Disease-specific survival (DSS) analysis stratified by ANK2 mutation status in LUAD from cBioportal

Using the cBioportal database, we first evaluated the ANK2 mutation frequency across multiple cancers. As shown in Fig. 2A, LUAD ranked second among all cancers, with a mutation frequency of 19.61%, followed by stomach adenocarcinoma and endometrial carcinoma. The distribution of various ANK2 mutation subtypes in LUAD was depicted in Fig. 2B, among which G1252V/R was the most prevalent. To investigate the influence of ANK2 mutation on LUAD patient survival, 12 studies from cBioportal with a total of 4309 LUAD patients, mainly receiving surgery and chemotherapy, were subjected to survival analysis. The results showed that the median overall survival (OS) (66.64 vs. 54.00 months, HR 0.99, 95% CI 0.76–1.29) (Fig. 2C), median PFS (35.84 vs. 36.13 months, HR 0.91, 95% CI 0.65–1.28) (Fig. 2D), and median disease-specific survival (DSS) (66.64 vs. 88.14 months, HR 0.92, 95%CI 0.58–1.46) (Fig. 2E) were comparable between LUAD patients with ANK2-MT and ANK2-WT (all P > 0.05).

### Association of ANK2 mutation with survival of LUAD patients treated with ICIs

**Fig. 3:**
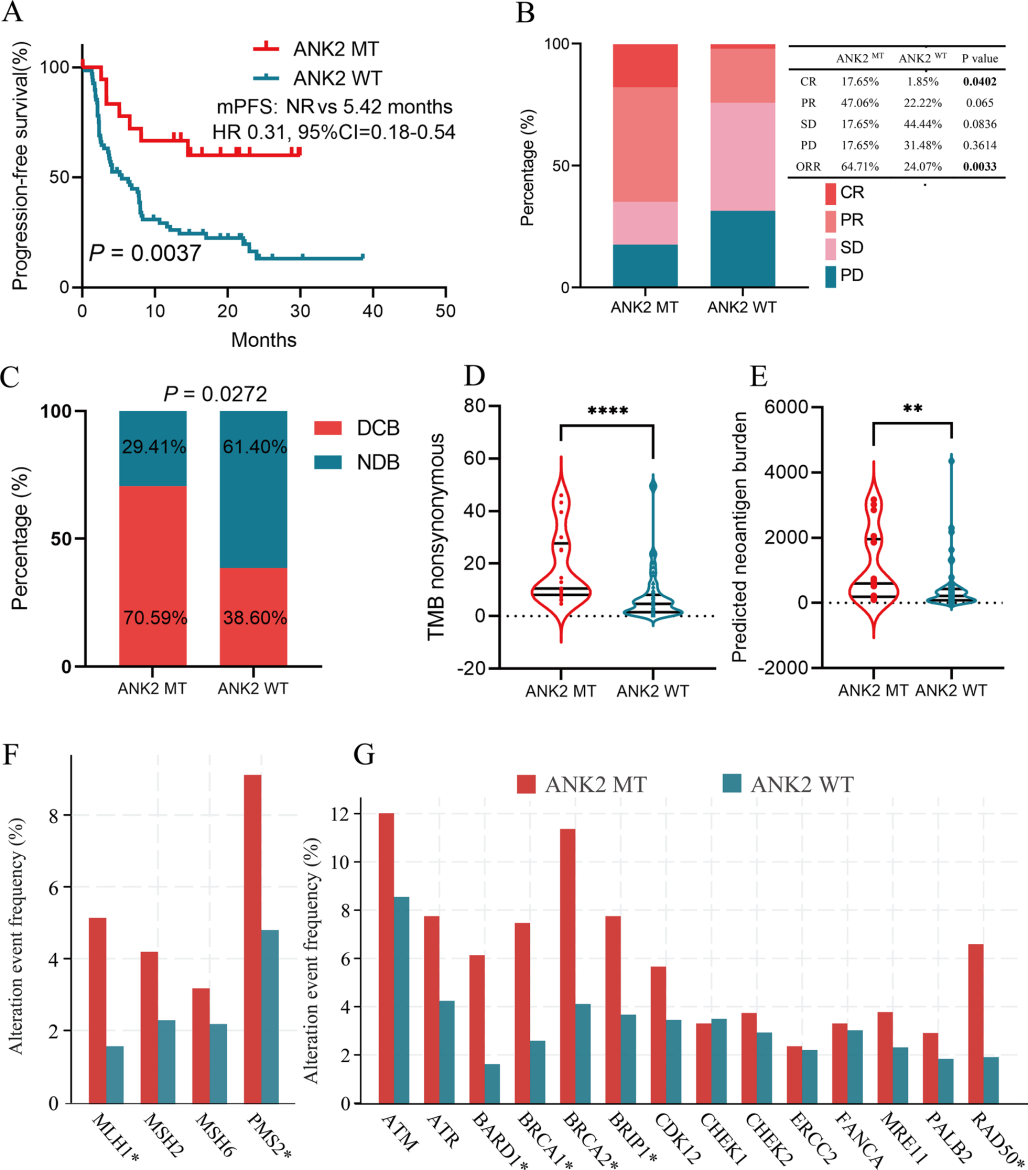
Association of ANK2 mutation with clinical outcomes in ICIs-treated LUAD cohort. **A**: Kaplan–Meier survival curves comparing PFS between the ANK2-MT and ANK2-WT LUAD in ICIs-treated cohort. **B**: Comparison of the proportion of patients with complete response (CR), partial response (PR), stable disease (SD) and progression disease (PD) between ANK2-MT and ANK2-WT LUAD in ICIs-treated cohort. **C**: Comparison of the proportion of patients with durable clinical benefit (DCB) or no durable benefit (NDB) between ANK2-MT and ANK2-WT LUAD in ICIs-treated cohort. **D**: Comparison of the TMB between ANK2-MT and ANK2-WT LUAD in ICIs-treated cohort. **E**: Comparison of the PNB between ANK2-MT and ANK2-WT LUAD in ICIs-treated cohort. **F**: The landscape of co-mutation between ANK2 and MMR pathway-related genes in LUAD. **G**: The landscape of co-mutation between ANK2 and DDR pathway-related genes in LUAD (NR, not reached; *P < 0.05, **P < 0.01, ****P < 0.0001)

To further investigate the impact of ANK2 mutation on ICIs efficacy in LUAD patients, a ICIs-treated cohort (Hellmann et al. and Rizvi et al.) from the cBioportal database was analyzed. The clinical characteristics of patients were summarized in Table 1. ANK2 mutated higher in LUAD patients with age ≥ 65 years than age < 65 years (P = 0.028), while ANK2 mutation frequency was comparable for males and females or smokers and non-smokers (all P > 0.05). Survival analysis revealed that PFS was significantly prolonged in patients with ANK2-MT than those with ANK2-WT (median PFS: NR vs. 5.42 months, HR 0.31, 95% CI 0.18–0.54, P = 0.0037) (Fig. 3A). Meanwhile, LUAD patients harboring ANK2-MT had significantly higher CR (17.65% vs. 1.85%, P = 0.0402), ORR (64.71% vs. 24.07%, P = 0.0033), and DCB (70.59% vs. 38.60%, P = 0.0272) (Fig. 3B, C) compared to their WT counterparts. In addition, tumor immunogenicity between ANK2-MT and ANK2-WT LUAD were compared. Higher TMB and PNB were observed in ANK2-MT LUAD (all P < 0.01) (Fig. 3D, E). The proportion of PD-L1-strong patients was higher in ANK2-MT LUAD than in ANK2-WT LUAD (21.1% vs. 17.6%) (Additional file 1: Fig. S1A). Notably, MMR-related genes (MLH1, PMS2) and DDR-related genes (BARD1, BRCA1, BRCA2, BRIP1, and RAD50) mutated more frequently in the ANK2-MT than in the ANK2-WT (all P < 0.05) (Fig. 3F, G). Furthermore, the frequency of co-mutations with ANK2 was analyzed. Multiple genes positively associated with ICIs efficacy, including LRP1B, KRAS, EPHA7, NOTCH4, and POLD1, had significantly higher mutational frequencies in ANK2-MT LUAD than in ANK2-WT LUAD (all P < 0.05) (Additional file 1: Fig. S1B). Conversely, lower frequency of EGFR mutation was observed in ANK2-MT LUAD (P < 0.05) (Additional file 1: Fig. S1C). The detailed mutation frequencies for each gene were summarized in Additional file 1: Table S1.

**Table 1:** Patient characteristics in the ICIs-treated LUAD cohort

Variables	ANK2 mutation (n = 17)(%)	Wild type (n = 57)(%)	P
Age			**0.028**
< 65	3 (17.6)	27 (47.4)	
≥ 65	14 (82.4)	30 (52.6)	
Sex			0.132
Female	13 (76.5)	32 (56.1)	
Male	4 (23.5)	25 (43.9)	
Smoking status			0.118
Current/former	16 (94.1)	44 (77.2)	
Never	1 (5.9)	13 (22.8)	

Bold value indicates P < 0.05

### Correlation between ANK2 mutation and TIME in LUAD

**Fig. 4:**
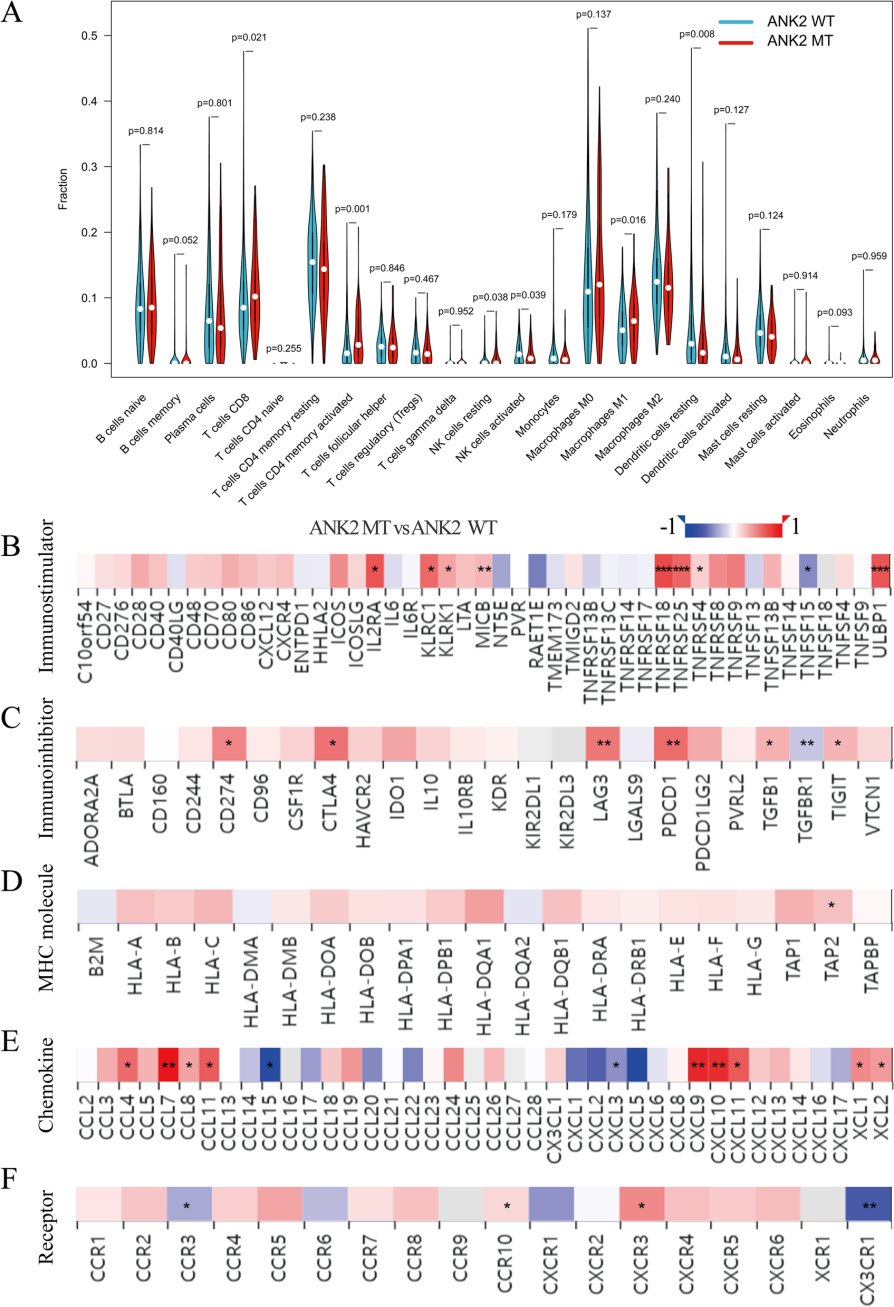
Association of ANK2 mutation with immune-related signatures in LUAD. **A**: Differences in immune cell infiltration between ANK2-MT and ANK2-WT LUAD. **B, C**: Differences in immuno-stimulators (**B**) and immuno-inhibitors (**C**) between ANK2-MT and ANK2-WT LUAD. **D**: Correlation of ANK2 mutation with the expression levels of MHC molecules in LUAD. **E, F**: The effect of ANK2 mutation on the expression of chemokines (**E**) and their receptors (**F**) in LUAD (*P < 0.05, **P < 0.01, ***P < 0.001)

Given the growing evidence on the pivotal role of TIME in ICIs efficacy, the correlation between ANK2 mutation and TIME in LUAD was investigated. The ANK2-MT LUAD was characterized by higher immune cell infiltration, including CD8^+^ T cells (P = 0.021), activated memory CD4^+^ T cells (P = 0.001), and M1 macrophages (P = 0.016) (Fig. 4A). TIMER database was used for further verification and similar results were obtained (Additional file 1: Fig. S2). Additionally, multiple “immuno-stimulator” genes were found to be much more abundant in the ANK2-MT LUAD than in the ANK2-WT LUAD, including IL2RA, KLRC1, KLRK1, MICB, TNFRSF18, TNFRSF25, TNFRSF4 and ULBP1 (all P < 0.05) (Fig. 4B). Furthermore, CD274, which encodes PD-L1 and is a predictive biomarker for increased ICIs efficacy, was higher in LUAD with ANK2-MT than with ANK2-WT (P < 0.05) (Fig. 4C). Several other immune checkpoints, including CTLA4, LAG3, PDCD1, TGFB1, and TIGIT, were upregulated in ANK2-MT LUAD as well (P < 0.05) (Fig. 4C). As shown in Fig. 4D, MHC molecules, particularly TAP2, were upregulated in ANK2-MT LUAD (P < 0.05). Besides, most chemokines (such as CXCL9, CXCL10, CXCL11 and XCL1) were more abundant in ANK2-MT LUAD, while CCL15 and CXCL3 expression was increased in ANK2-WT LUAD (all P < 0.05) (Fig. 4E). In terms of chemokine receptors, CCR10 and CXCR3 was upregulated in ANK2-MT LUAD; while CCR3 and CX3CR1, on the other hand, were downregulated (all P < 0.05) (Fig. 4F).

### Association of ANK2 expression with clinical features and survival of LUAD patients

**Fig. 5:**
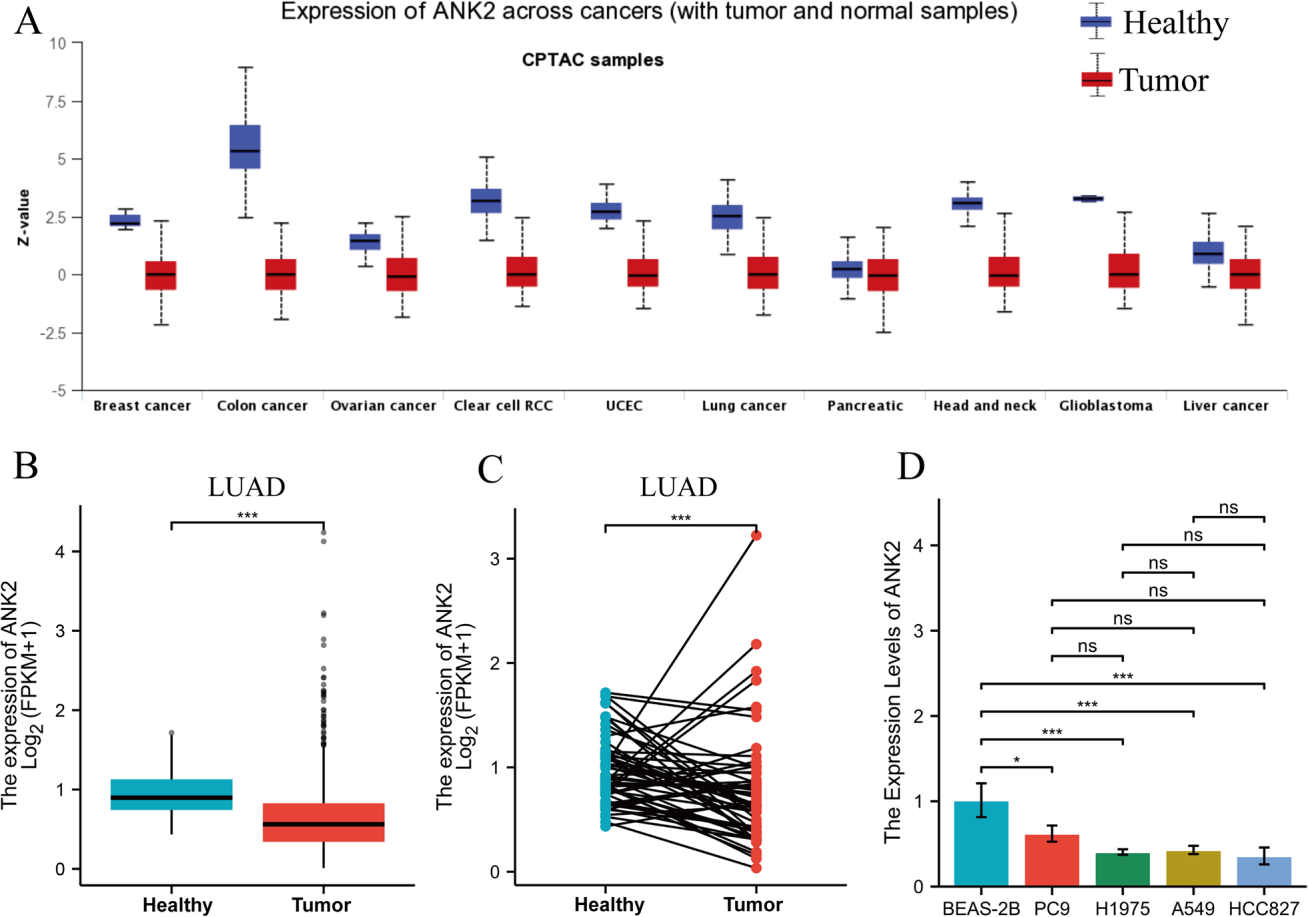
Comparison of ANK2 expression in healthy tissue versus tumor tissue. **A**: ANK2 expression levels in pan-cancer were measured using UALCA database. **B**: Comparison of ANK2 expression between LUAD and non-paired healthy tissues in TCGA. **C**: Comparison of ANK2 expression between LUAD and paired healthy tissues in TCGA. **D**: Comparison of ANK2 expression between healthy lung epithelial cell line (BEAS-2B) and LUAD cell lines (PC9, H1975, A549, and HCC827) (*P < 0.05, ***P < 0.001)

**Fig. 6:**
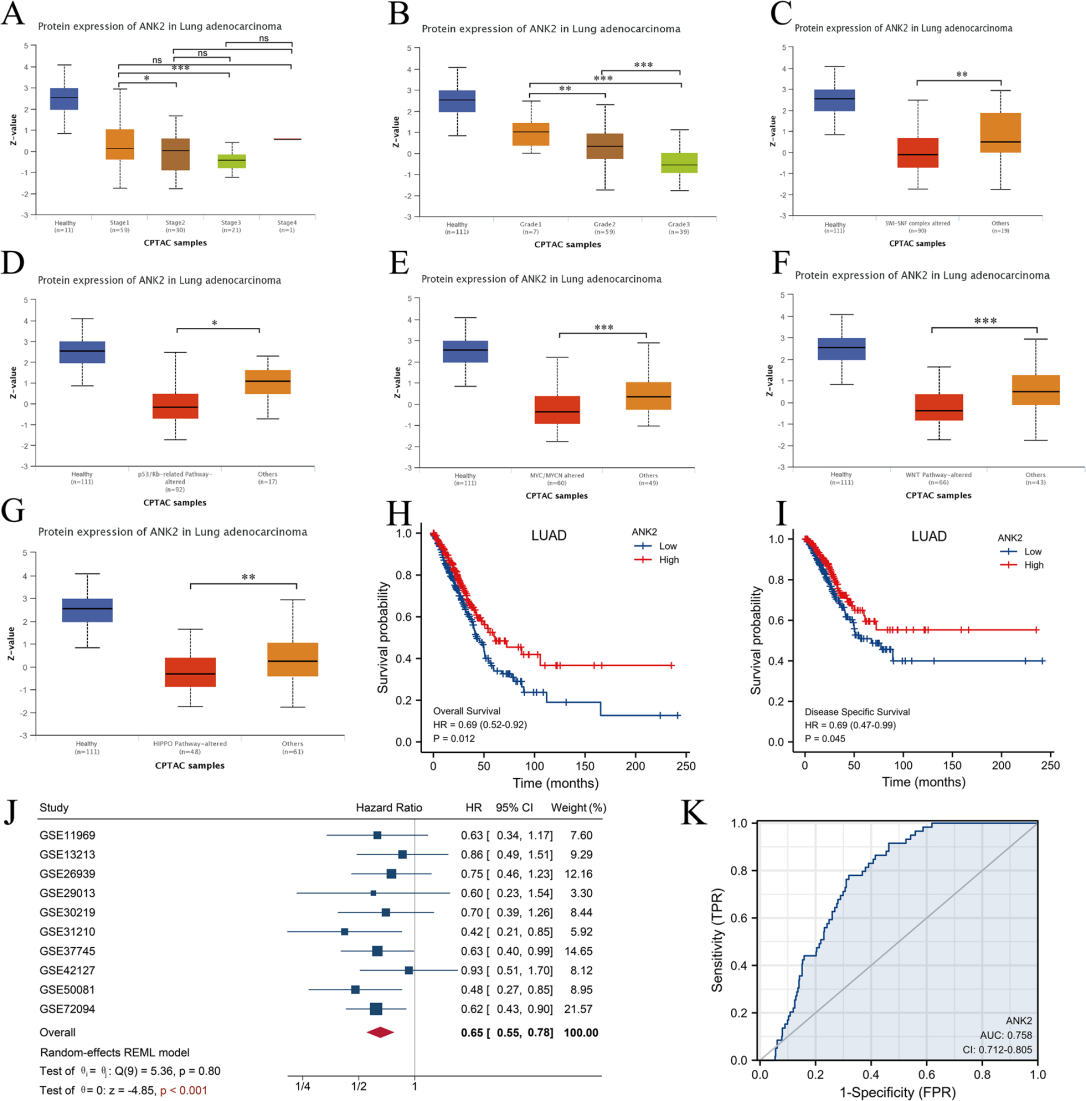
Association of ANK2 expression with clinical features and survival of LUAD patients **A**: Pathological stage. **B**: Histological grade. **C**: SWI-SNF complex alteration. **D**: P53/Rb-related pathway alteration. **E**: MYC/MYCN alteration. **F**: WNT pathway alteration. **G**: HIPPO pathway alteration. **H**: Kaplan–Meier curves for OS of patients in the high- and low-ANK2 expression LUAD. **I**: Kaplan–Meier curves for DSS of patients in the high- and low-ANK2 expression LUAD. **J**: Meta-analysis was performed to integrate HR values about ANK2 expression and OS from ten GEO LUAD cohorts. **K**: Diagnostic ROC curve analysis of ANK2 in LUAD (*P < 0.05, **P < 0.01, ***P < 0.001)

Firstly, ANK2 expression was compared between tumor tissues and healthy tissues. As shown in Fig. 5A, the CPTAC database revealed that ANK2 expression was lower compared to healthy tissues across multiple cancers, including lung cancer. Likewise, compared with healthy tissue, LUAD expressed lower ANK2 expression in TCGA database (P < 0.001) (Fig. 5B, C). For further verification, q-PCR was performed. ANK2 expression was significantly lower in lung adenocarcinoma cell lines (PC9, H1975, A549 and HCC827, all P < 0.05) than in healthy lung epithelial cell line (BEAS-2B) (Fig. 5D). Secondly, correlation between ANK2 expression and multiple clinical characteristics was analyzed using TCGA data. It was observed that higher ANK2 expression at mRNA level correlated with earlier T-stage, earlier N-stage, earlier pathological stage, and fewer dead events (all P < 0.05) (Table 2). CPTAC database further validated that higher ANK2 expression at protein level was associated with earlier pathological stage (P < 0.05) (Fig. 6A) and lower histological grade (P < 0.01) (Fig. 6B). Moreover, we found that ANK2 expression was decreased in LUAD harboring SWI-SNF complex alteration, P53/Rb-related pathway alteration, MYC alteration, WNT pathway alteration, and HIPPO pathway alteration (all P < 0.05) (Fig. 6C–G), all of which have been shown to play important roles in tumorigenesis. Next, survival analyses were conducted to investigate the prognostic value of ANK2 expression in LUAD. Notably, patients with higher ANK2 expression experienced longer OS (P = 0.012, HR = 0.69, 95% CI 0.52–0.92) (Fig. 6H) and longer DSS (P = 0.045, HR = 0.69, 95% CI 0.47–0.99) (Fig. 6I). The meta-analysis of ten GEO LUAD cohorts showed a pooled HR of 0.65 (0.55–0.78) between high ANK2 expression and OS (Fig. 6J), further confirming the protective role of ANK2 in LUAD. The ROC curve demonstrated that the diagnosticity of ANK2 was excellent with an AUC of 0.758 (Fig. 6K).

**Table 2:** Association between ANK2 mRNA expression and clinical characteristics in LUAD

Characteristic	Low expression of ANK2	High expression of ANK2	P
n	267	268	
T stage, n (%)			** < 0.001**
T1	66 (24.8%)	109 (41%)	
T2	157 (59%)	132 (49.6%)	
T3	30 (11.3%)	19 (7.1%)	
T4	13 (4.9%)	6 (2.3%)	
N stage, n (%)			**0.025**
N0	162 (61.6%)	186 (72.7%)	
N1	53 (20.2%)	42 (16.4%)	
N2	47 (17.9%)	27 (10.5%)	
N3	1 (0.4%)	1 (0.4%)	
M stage, n (%)			0.357
M0	189 (92.2%)	172 (95%)	
M1	16 (7.8%)	9 (5%)	
Pathologic stage, n (%)			**0.015**
Stage I	132 (49.8%)	162 (61.8%)	
Stage II	64 (24.2%)	59 (22.5%)	
Stage III	53 (20%)	31 (11.8%)	
Stage IV	16 (6%)	10 (3.8%)	
Gender, n (%)			0.280
Female	136 (50.9%)	150 (56%)	
Male	131 (49.1%)	118 (44%)	
Age, n (%)			0.187
< = 65	135 (52.5%)	120 (46.3%)	
> 65	122 (47.5%)	139 (53.7%)	
Smoker, n (%)			0.630
No	35 (13.5%)	40 (15.3%)	
Yes	225 (86.5%)	221 (84.7%)	
Primary therapy outcome, n (%)			0.544
PD	37 (17.2%)	34 (14.7%)	
SD	20 (9.3%)	17 (7.4%)	
PR	4 (1.9%)	2 (0.9%)	
CR	154 (71.6%)	178 (77.1%)	
OS event, n (%)			**0.003**
Alive	154 (57.7%)	189 (70.5%)	
Dead	113 (42.3%)	79 (29.5%)	

Bold values indicate P < 0.05

### Correlation between ANK2 expression and TIME in LUAD

**Fig. 7:**
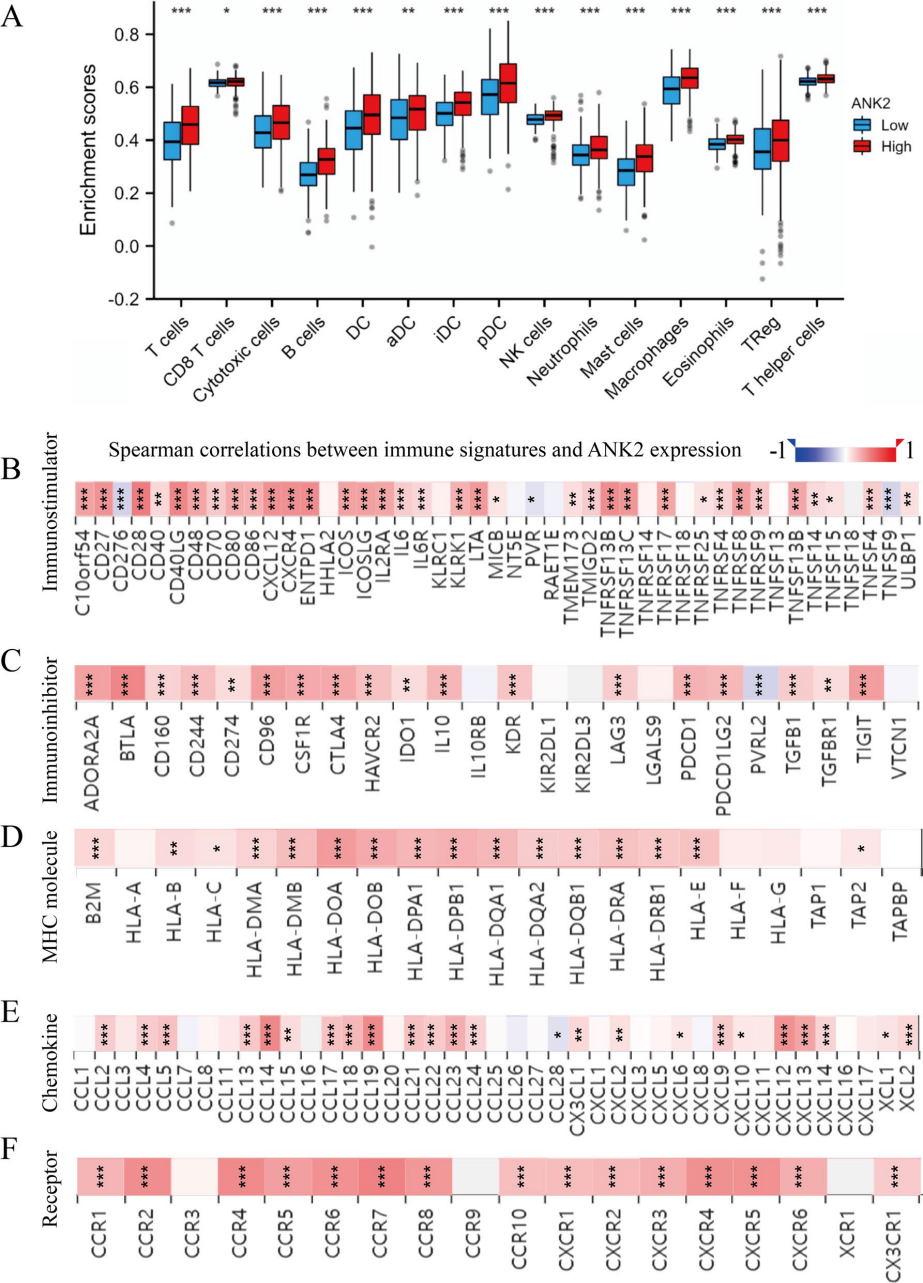
Association between ANK2 expression and immune-related signatures in LUAD **A**: Differences in immune cell infiltration between high- and low-ANK2 expression LUAD. **B, C**: Association of ANK2 expression with immuno-stimulators (**B**) and immuno-inhibitors (**C**) in LUAD. **D**: Correlation of ANK2 expression with MHC molecules expression in LUAD. **E, F**: Correlation of ANK2 expression with the expression of chemokines (**E**) and their receptors (**F**) in LUAD (*P < 0.05, **P < 0.01, ***P < 0.001)

The correlation between ANK2 expression and immune cell infiltration was further explored. The results showed that increased ANK2 expression correlated with increased infiltration of a variety of immune cells, including T cells, CD8^+^ T cells, cytotoxic T cells, B cells, DC, aDC, iDC, pDC, NK cells, neutrophils, mast cells, macrophages, eosinophils, Tregs, and T helper cells (all P < 0.05) (Fig. 7A). Using the TIMER database, positive correlations were further established between ANK2 expression and infiltration of immune cells (CD4^+^ T cells, CD8^+^ T cells, B cells, macrophages, DC, and neutrophils) (Additional file 1: Fig. S3A). Meanwhile, significant positive correlations were identified between ANK2 expression and molecular biomarkers of multiple immune cells, including T cells, CD8^+^ T cells, Tfh, NK cells, and dendritic cells (all P < 0.05) (Table 3). Notably, ANK2 copy number was correlated with immune cell infiltration as well, with a higher level of immune cell infiltration in ANK2 arm-level gain LUAD compared to ANK2 arm-level deletion LUAD (Additional file 1: Fig. S3B). Moreover, LUAD with high ANK2 expression was more abundant in C2 (IFN-gamma dominant) and C3 (inflammatory) immune subtypes (P < 0.001) (Additional file 1: Fig. S3C).

**Table 3:** Correlation analysis of ANK2 and marker s of multiple immune cells in LUAD by TIMER

Description	Gene markers	rho	P value
T cell	CD3D	0.342	1.50E−15
	CD3E	0.442	4.77E−26
	CD2	0.391	3.27E−20
CD8^+^ T cell	CD8A	0.316	2.05E−13
	CD8B	0.268	6.18E−10
	IL2RA	0.362	2.41E−17
Tfh	CXCR3	0.367	7.08E−18
	CXCR5	0.47	1.06E−29
	ICOS	0.417	4.14E−23
Th1	IL12RB1	0.404	1.21E−21
	CCR1	0.34	2.04E−15
	CCR5	0.425	5.84E−24
Th2	CCR4	0.474	3.14E−30
	CCR8	0.426	4.48E−24
	HAVCR1	0.04	3.62E−01
Th17	IL21R	0.4	3.03E−21
	IL23R	0.306	1.37E−12
	CCR6	0.483	1.69E−31
B cell	CD19	0.455	1.01E−27
	CD79A	0.429	1.77E−24
Monocyte	CD86	0.36	3.28E−17
	CSF1R	0.385	1.31E−19
Natural killer cell	KIR2DL1	0.143	1.11E−03
	KIR2DL3	0.167	1.16E−04
	KIR2DL4	0.079	7.31E−02
	KIR3DL1	0.081	6.65E−02
	KIR3DL2	0.182	3.26E−05
	KIR2DS4	0.156	3.86E−04
Dendritic cell	HLA-DPB1	0.36	3.41E−17
	HLA-DQB1	0.262	1.49E−09
	HLA-DRA	0.299	4.10E−12
	HLA-DPA1	0.342	1.28E−15
	CD1C	0.312	4.20E−13
	NRP1	0.247	1.28E−08
	ITGAX(CD11C)	0.402	2.22E−21

Immuno-stimulators, immuno-inhibitors, MHC molecules, chemokine and their receptors are essential components for immune response. Therefore, the association of these signatures with ANK2 expression was evaluated. Most of the immuno-stimulators, immune checkpoint molecules and MHC molecules were positively correlated with ANK2 expression (most P < 0.05) (Fig. 7B–D). In terms of chemokines and their receptors, significantly positive correlations with ANK2 expression were detected (most P < 0.05) (Fig. 7E, F). Taken together, these results suggested that high ANK2 expression was associated with immunologically “hot” tumor microenvironment and enhanced anti-tumor immunity.

## Discussion

ICIs treatment has revolutionized the treatment of lung cancer including LUAD, but disappointingly only a subset of patients responds to ICIs. Identifying novel biomarkers are imperative. In this study, we revealed that ANK2 was a predictive biomarker for ICIs efficacy in LUAD patients, with individuals harboring ANK2 mutation experiencing longer PFS. Moreover, high ANK2 expression correlated with prolonged survival for LUAD patients. Importantly, ANK2 mutation or high ANK2 expression was associated with the development of enhanced anti-tumor immunity.

The gene mutation status in LUAD guides the therapeutic decision-making, with targeted therapy being the first choice for patients harboring EGFR or ALK mutations [14]. Of note, a growing number of studies demonstrate that certain gene mutations have profound impacts on the efficacy of immunotherapy [15–17]. In our study, ANK2 mutation was first identified to be associated with longer PFS and higher response rates for patients receiving ICIs, indicating a favorable effect of ANK2 mutation on immunotherapy. LUAD patients with EGFR mutation rarely benefit from ICIs [42], which could be attributed to the immune-inert phenotype of EGFR mutated LUAD [43]. In contrast, ANK2-MT LUAD exhibited higher levels of TMB and PD-L1 expression, providing stronger evidence that ANK2 mutation support a better survival benefit from ICIs for LUAD patients. In clinical practice, precisely identifying the dominant benefiting population from ICIs is essential but challenging. ANK2, with a high mutation frequency in LUAD, could be a promising biomarker given the increased efficacy for ICIs.

An activated cancer-immunity cycle is indispensable for the success of ICIs [44, 45], with antigen release being the critical first step [44]. In our study, high PNB were observed in the ANK2-MT LUAD, indicating that the ANK2-MT LUAD had heightened tumor immunogenicity [46]. In addition, higher mutation frequencies of MMR-related genes, DDR-related genes and POLD1 were found in ANK2-MT LUAD, which were reported to be positively correlated with increased immunogenicity [47–49]. Therefore, ANK2 mutation might enhance the immunogenicity of LUAD, hence boosting the cancer-immunity cycle. Presenting tumor antigens to T cells is the second step of cancer-immunity cycle [44, 50] and our results revealed that in ANK2-MT LUAD, DCs and MHC molecules, two key participants in antigen-presenting [51, 52], were elevated. The third step of the cancer immunity cycle is the trafficking of activated effector T cells into TIME, which guarantees their anti-tumor immunity [44]. ANK2-MT LUAD was characterized by high expression of chemokines (CXCL9, CXCL10, CXCL11, and CXCR3) involved in the recruitment of anti-cancer immune cells [53]. Consequently, ANK2-MT LUAD exhibited a TIME characterized by increased anti-tumor immune cells.

Concerning ANK2 expression, it has been found to be downregulated in breast cancer and colon adenocarcinoma [30, 54], which was consistent with the findings of our pan-cancer analysis. In terms of lung cancer, ANK2 has not been studied and we proved that ANK2 was downregulated in LUAD. As we all know, the type of TIME plays an important role in the elimination of cancer cells [55, 56], with “hot” TIME facilitating cancer cell killing. According to our findings, ANK2 expression was strongly associated with multiple immune cell infiltration, promoting the formation of "hot" TIME. Moreover, positive correlations were observed between ANK2 expression and immune-related signatures (MHC molecules, immuno-stimulators, immuno-inhibitors, chemokines and their receptors), with the majority of these profiles enhancing antitumor TIME [55, 57, 58]. These findings suggested that LUAD patients with high ANK2 expression had improved antitumor immunity and enhanced "hot" TIME, which provided explanations for a better prognosis of these patients. Thus, it could be speculated that ANK2 agonists are likely to promote antitumor immunity and thus increase the efficacy of immunotherapy. Given the similar impact of ANK2 mutation and high ANK2 expression on TIME of LUAD, we conjectured that ANK2 mutation might alter ANK2 expression, however, statistical analysis revealed no correlation between ANK2 mutation and ANK2 expression (Additional file 1: Fig. S4).

Although we have systematically and comprehensively explored the predictive and prognostic roles of ANK2 in LUAD, some non-negligible limitations exist in our study. Firstly, the limited sample size and the absence of OS data for ICIs-treated cohort could bias the role of ANK2 mutation in LUAD patients receiving immunotherapy. Secondly, the potential role of ANK2 in TIME was analyzed based on the LUAD samples from public databases where clinical characteristics of patients were likely to be highly heterogeneous, which could influence the reliability of the findings. Further validation utilizing in vitro experiments and in vivo animal models is required. Thirdly, there is a lack of direct evidence on the relationship between ANK2 expression and prognosis of patients with LUAD treated with ICIs. Notwithstanding, our study provided comprehensively predictive and prognostic evidence concerning ANK2 in LUAD for the first time, particularly the favorable significance of ANK2 mutation in LUAD patients treated with ICIs, paving the way for future molecular studies on the role of ANK2 in LUAD.

## Conclusion

Our study was the first to propose that ANK2 mutation correlated with prolonged PFS and enhanced response for LUAD patients receiving ICIs therapy. LUAD patients with higher ANK2 expression experienced more favorable outcomes. In conclusion, it was demonstrated that ANK2 (ANK2 mutation or high ANK2 expression) was potentially favorable biomarker for LUAD prognosis. Moreover, ANK2 (ANK2 mutation or high ANK2 expression) is related to strengthened tumor immunogenicity and inflamed antitumor immunity, which could be the underlying mechanism for the function of ANK2 in LUAD.

## Supplementary Information

**Additional file1**.** Figure S1** Relationship between ANK2 mutation and clinical features in LUAD.** A**: ANK2 mutation was associated with enhanced PD-L1 expression in LUAD.** B**: Frequency of gene mutations with positive effects on immunotherapy in ANK2-MT and ANK2-WT LUAD.** C**: Frequency of gene mutations with negative effects on immunotherapy in ANK2-MT and ANK2-WT LUAD (*P < 0.05).** Figure S2** Comparison of immune infiltration between ANK2-MT and ANK2-WT LUAD from TIMER database (*P < 0.05).** Figure S3** Association of ANK2 expression with immune infiltration in LUAD.** A**: Correlation of ANK2 expression with immune infiltration in LUAD patients from TIMER database.** B**: ANK2 copy number variation affects the infiltrating levels of CD8^+^ T cell, CD4^+^ T cell, B cell, dendritic cell, macrophages, and neutrophils in LUAD.** C**: ANK2 expression in different immune subtypes in LUAD via TISIDB (*P < 0.05, **P < 0.01, ***P < 0.001).** Figure S4** Relationship between ANK2 mutation and ANK2 expression in LUAD.

## Data Availability

This study was conducted based on publicly available databases, including TCGA (https://portal.gdc.cancer.gov/repository?facetTab=cases), GEO (https://www.ncbi.nlm.nih.gov/geo/), TIMER (https://cistrome.shinyapps.io/timer/), UALCAN (http://ualcan.path.uab.edu/), TISIDB (http://cis.hku.hk/TISIDB/index.php) and cBioportal (https://www.cbioportal.org/). The detailed databases used in the study were described in the method section.

## Abbreviations

ANK2: Ankyrin-2
ANK2-MT: ANK2-mutant
ANK2-WT: ANK2 wild type
ALK: Anaplastic lymphoma kinase
CR: Complete response
CTLA-4: Cytotoxic T lymphocyte-associated antigen 4
DCB: Durable clinical benefit
DDR: DNA damage repair
DFS: Disease free survival
DSS: Disease specific survival
EGFR: Epidermal growth factor receptor
GEO: Gene Expression Omnibus
ICIs: Immune checkpoint inhibitors
LUAD: Lung adenocarcinoma
MHC: Major histocompatibility complex
MMR: Mismatch repair
NDB: No durable benefit
NSCLC: Non-small cell lung cancer
ORR: Objective response rate
OS: Overall survival
PD: Progressive disease
PD-1: Programmed cell death protein 1
PD-L1: Programmed cell death protein ligand 1
PFS: Progression free survival
PNB: Predicted neoantigen burden
PR: Partial response
q-PCR: Quantitative polymerase chain reaction
SD: Stable disease
ssGSEA: Single-sample gene set enrichment analysis
TCGA: The Cancer Genome Atlas
TCR: T cell receptor
TIME: Tumor immune microenvironment
TMB: Tumor mutation burden
