# Supplementary material for: ANK2 as a novel predictive biomarker for immune checkpoint inhibitors and its correlation with antitumor immunity in lung adenocarcinoma
Source: BMC Pulm Med. 2022 Dec 20;22:483. doi: 10.1186/s12890-022-02279-2 (PMC9768990; doi:10.1186/s12890-022-02279-2)
Supplement: Supplementary file 1 — Additional file1. Figure S1 Relationship between ANK2 mutation and clinical features in LUAD. A: ANK2 mutation was associated with enhanced PD-L1 expression in LUAD. B: Frequency of gene mutations with positive effects on immunotherapy in ANK2-MT and ANK2-WT LUAD. C: Frequency of gene mutations with negative effects on immunotherapy in ANK2-MT and ANK2-WT LUAD (*P < 0.05). Figure S2 Comparison of immune infiltration between ANK2-MT and ANK2-WT LUAD from TIMER database (*P < 0.05). Figure S3 Association of ANK2 expression with immune infiltration in LUAD. A: Correlation of ANK2 expression with immune infiltration in LUAD patients from TIMER database. B: ANK2 copy number variation affects the infiltrating levels of CD8+ T cell, CD4+ T cell, B cell, dendritic cell, macrophages, and neutrophils in LUAD. C: ANK2 expression in different immune subtypes in LUAD via TISIDB (*P < 0.05, **P < 0.01, ***P < 0.001). Figure S4 Relationship between ANK2 mutation and ANK2 expression in LUAD. [file 12890_2022_2279_MOESM1_ESM.docx]

***Supplementary material***

**
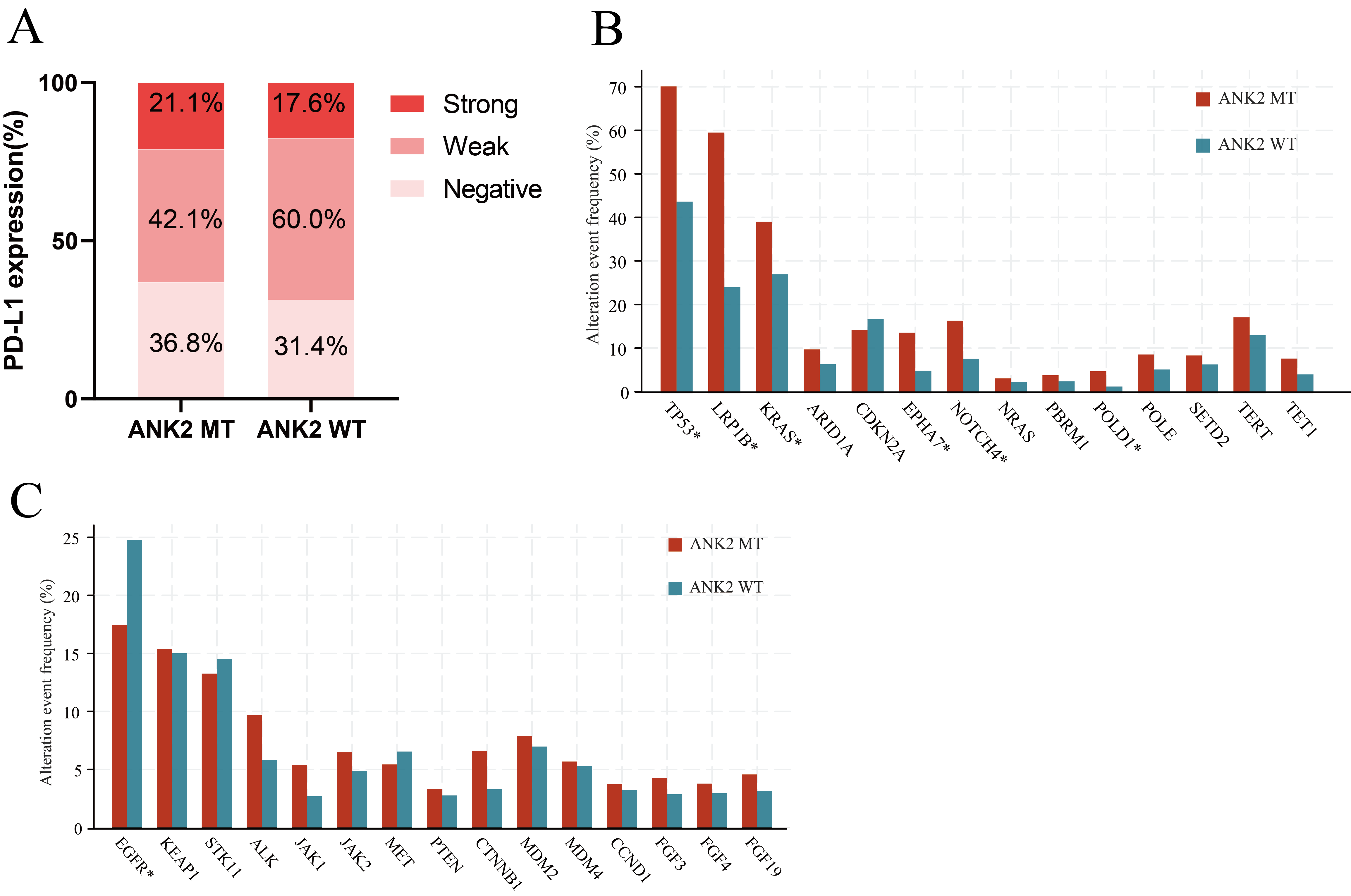
**

**Figure S1| Relationship between ANK2 mutation and clinical features in LUAD.** **A**: ANK2 mutation was associated with enhanced PD-L1 expression in LUAD. **B**: Frequency of gene mutations with positive effects on immunotherapy in ANK2-MT and ANK2-WT LUAD. **C**: Frequency of gene mutations with negative effects on immunotherapy in ANK2-MT and ANK2-WT LUAD (*P < 0.05).


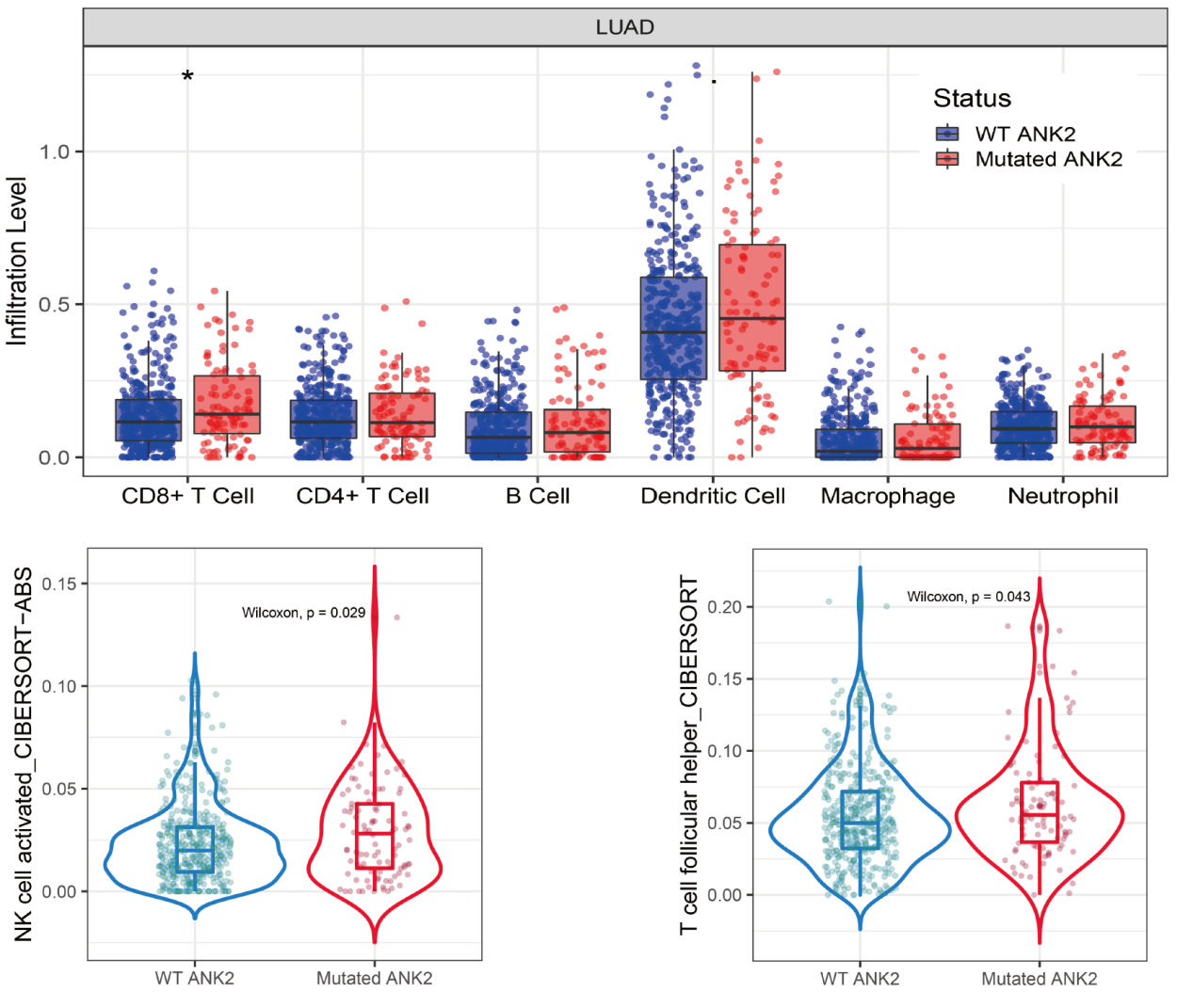


**Figure S2| Comparison of immune infiltration between ANK2-MT and ANK2-WT LUAD** **from TIMER database** (*P < 0.05).

**
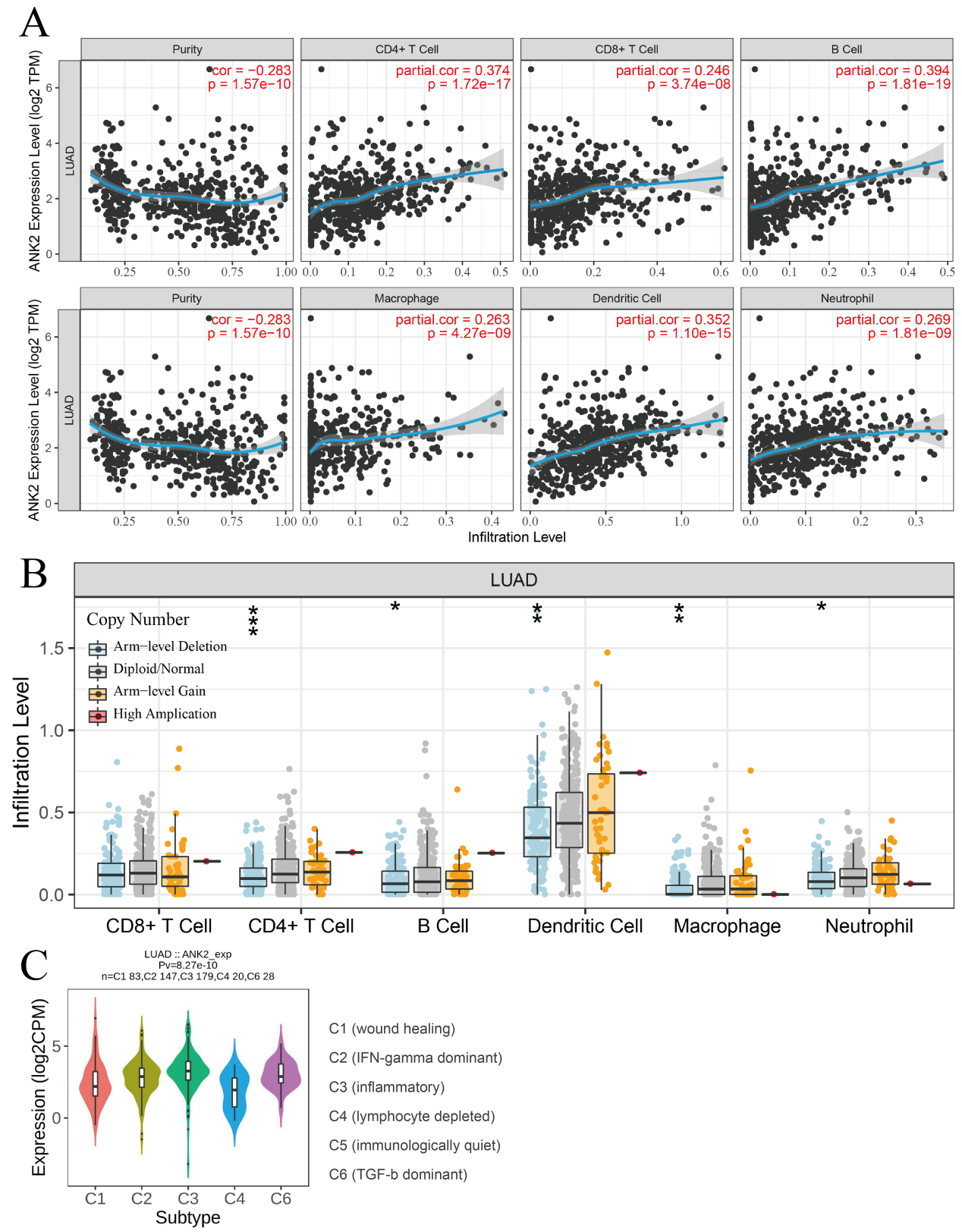
**

**Figure S3| Association of ANK2 expression with immune infiltration in LUAD. A:** Correlation of ANK2 expression with immune infiltration in LUAD patients from TIMER database. **B:** ANK2 copy number variation affects the infiltrating levels of CD8^+^ T cell, CD4^+^ T cell, B cell, dendritic cell, macrophages, and neutrophils in LUAD. **C:** ANK2 expression in different immune subtypes in LUAD via TISIDB (*P < 0.05, **P < 0.01, ***P < 0.001).

**
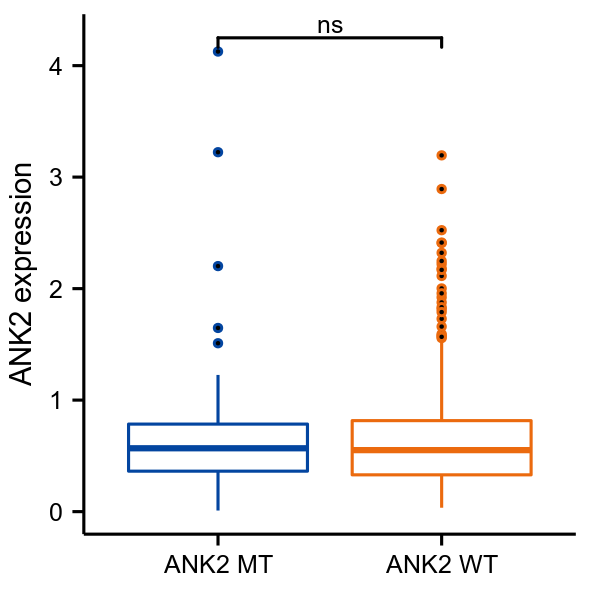
**

**Figure S4| Relationship between ANK2 mutation and ANK2 expression in LUAD.**

**Table S1**

| Statistical analysis of MMR gene co-mutation with ANK2 | | | | |
| --- | --- | --- | --- | --- |
| Gene | ANK2 MT | ANK2 WT | P value | Q value |
| MLH1 | 5.19（11/212） | 1.61（22/1363） | 2.61E-03 | 0.021 |
| MSH2 | 4.25(9/212) | 2.34(24/1026) | 0.0955 | 0.211 |
| MSH6 | 3.23(6/186) | 2.23(29/1299) | 0.268 | 0.386 |
| PMS2 | 9.17(21/229) | 4.85(63/1299) | 9.20E-03 | 0.0494 |
|  |  |  |  |  |
| Statistical analysis of DDR gene co-mutation with ANK2. | | | | |
| Gene | ANK2 MT | ANK2 WT | P value | Q value |
| ATM | 12.02（31/258） | 8.55（119/1392） | 0.518 | 0.145 |
| ATR | 7.75（20/258） | 4.24（59/1392） | 0.0153 | 0.0685 |
| BARD1 | 6.13（13/212） | 1.62（21/1299） | 3.47E-04 | 5.13E-03 |
| BRCA1 | 7.47(18/241) | 2.59(36/1392) | 3.91E-04 | 5.57E-03 |
| BRCA2 | 11.36(30/264) | 4.11(56/1363) | 1.06E-05 | 3.71E-04 |
| BRIP1 | 7.75(20/258) | 3.67(50/1363) | 4.42E-03 | 0.0305 |
| CDK12 | 5.66(12/212) | 3.45(47/1363) | 0.088 | 0.2 |
| CHEK1 | 3.30(7/212) | 3.49(39/1116) | 0.543 | 0.583 |
| CHEK2 | 3.73(9/241) | 2.93(38/1299) | 0.308 | 0.418 |
| ERCC2 | 2.36(5/212) | 2.20(28/1270) | 0.521 | 0.568 |
| FANCA | 3.3(7/212) | 3.02(42/1392) | 0.476 | 0.54 |
| MRE11 | 3.77(8/212) | 2.31(30/1299) | 0.152 | 0.2797 |
| PALB2 | 2.9(7/241) | 1.83(25/1363) | 0.194 | 0.318 |
| RAD50 | 6.59(17/258) | 1.91(26/363) | 1.35E-04 | 2.51E-03 |
|  |  |  |  |  |
| Statistical analysis of co-mutation between ANK2 mutation and gene mutations positively associated with immunotherapy efficacy | | | | |
| Gene | ANK2 MT | ANK2 WT | P value | Q value |
| ARID1A | 9.69(25/258) | 6.32(88/1392) | 0.0374 | 0.117 |
| CDKN2A | 14.17(35/247) | 16.67(232/1392) | 0.189 | 0.313 |
| EPHA7 | 13.54(31/229) | 4.81(67/1392) | 3.56E-06 | 1.57E-04 |
| KRAS | 39.02(103/264) | 26.94(375/1392) | 6.84E-05 | 1.52E-03 |
| LRP1B | 59.47(157/264) | 23.99(334/1392) | 1.61E-28 | 2.02E-24 |
| NOTCH4 | 16.28(42/258) | 7.56(103/1363) | 2.19E-05 | 6.44E-04 |
| NRAS | 3.06(7/229） | 2.20（28/1270） | 0.279 | 0.396 |
| PBRM1 | 3.73（9/241） | 2.37（33/1392） | 0.155 | 0.283 |
| POLD1 | 4.69（9/192) | 1.15(15/1299) | 1.86E-03 | 0.0167 |
| POLE | 8.53(22/258) | 5.08(66/1299) | 0.0245 | 0.0929 |
| STED2 | 8.3(20/241) | 6.25(87/1392) | 0.148 | 0.275 |
| TERT | 17.05(44/258) | 13.00(181/1392） | 0.0528 | 0.147 |
| TET1 | 7.58（20/264） | 3.95（55/1392） | 0.0105 | 0.0538 |
| TP53 | 70.08（85/264） | 43.61（60/1392） | 1.43E-05 | 2.06E-12 |
|  |  |  |  |  |
| Statistical analysis of co-mutation between ANK2 mutation and gene mutations negatively associated with immunotherapy efficacy | | | | |
| Gene | ANK2 MT | ANK2 WT | P value | Q value |
| EGFR | 17.44（45/258） | 24.78（345/1392） | 5.74E-03 | 0.0363 |
| KEAP1 | 15.38(38/247) | 15.01(209/1392) | 0.472 | 0.538 |
| STK11 | 13.26(35/264) | 14.5(202/1392) | 0.336 | 0.44 |
| ALK | 9.69(25/258) | 5.82(81/1392) | 0.0176 | 0.0751 |
| JAK1 | 5.39(13/241) | 2.69(35/1299) | 0.0278 | 0.0991 |
| JAK2 | 6.48(16/247) | 4.89(68/1392) | 0.185 | 0.309 |
| MET | 5.43（14/258） | 6.54(91/1392） | 0.304 | 0.415 |
| PTEN | 3.32（8/241） | 2.76（35/1270） | 0.377 | 0.473 |
| CTNNB1 | 6.60（14/212） | 3.30（46/1392） | 0.0205 | 0.0829 |
| MDM2 | 7.88（19/241） | 6.97（95/1363） | 0.346 | 0.449 |
| MDM4 | 5.68（13/229） | 5.28（72/1363） | 0.452 | 0.522 |
| CCND1 | 3.73（9/241） | 3.23（44/1363） | 0.401 | 0.487 |
| FGF3 | 4.26(11/258) | 2.87(40/1392) | 0.16 | 0.288 |
| FGF4 | 3.77(8/212) | 2.93(40/1363) | 0.314 | 0.423 |
| FGF19 | 4.56(11/241) | 3.15(43/1363) | 0.176 | 0.304 |
